# Mechanisms of Antibiotic Tolerance in *Mycobacterium avium* Complex: Lessons From Related Mycobacteria

**DOI:** 10.3389/fmicb.2020.573983

**Published:** 2020-09-30

**Authors:** Harley Parker, Rachel Lorenc, Jennie Ruelas Castillo, Petros C. Karakousis

**Affiliations:** ^1^Department of Medicine, Johns Hopkins University School of Medicine, Baltimore, MD, United States; ^2^Department of International Health, Johns Hopkins Bloomberg School of Public Health, Baltimore, MD, United States

**Keywords:** *Mycobacterium avium*, tuberculosis, antibiotic tolerance, persistence, models, macrophage, nutrient starvation, hypoxia

## Abstract

*Mycobacterium avium* complex (MAC) species are the most commonly isolated nontuberculous mycobacteria to cause pulmonary infections worldwide. The lengthy and complicated therapy required to cure lung disease due to MAC is at least in part due to the phenomenon of antibiotic tolerance. In this review, we will define antibiotic tolerance and contrast it with persistence and antibiotic resistance. We will discuss physiologically relevant stress conditions that induce altered metabolism and antibiotic tolerance in mycobacteria. Next, we will review general molecular mechanisms underlying bacterial antibiotic tolerance, particularly those described for MAC and related mycobacteria, including *Mycobacterium tuberculosis*, with a focus on genes containing significant sequence homology in MAC. An improved understanding of antibiotic tolerance mechanisms can lay the foundation for novel approaches to target antibiotic-tolerant mycobacteria, with the goal of shortening the duration of curative treatment and improving survival in patients with MAC.

## Introduction

*Mycobacterium avium* complex (MAC) comprises multiple genetically similar species that are often not differentiated in the clinical microbiology laboratory, including *M. avium, Mycobacterium intracellulare, Mycobacterium arosiense, Mycobacterium bouchedurhonense, Mycobacterium chimaera, Mycobacterium colombiense, Mycobacterium marseillense, Mycobacterium timonense, Mycobacterium vulneris, and Mycobacterium yongonense*. *Mycobacterium avium* complex species, and specifically *M. avium* and *M. intracellulare*, are the most common cause of pulmonary infection worldwide among the nontuberculous mycobacteria (NTM), and typically occurs in the setting of pre-existing lung disease. Disseminated MAC occurs primarily in patients with severe immune deficiency, such as those with advanced HIV infection, hematologic malignancy, or a history of immunosuppressive therapy, such as anti-tumor necrosis factor-α (TNF-α) therapy (Karakousis, 2009; Winthrop et al., 2009).

Unlike with *Mycobacterium tuberculosis*, there is no evidence of human-to-human transmission of MAC. Instead, MAC species are acquired from the environment, where they are ubiquitous as free-living organisms, including in municipal water sources, soil, and house dust (Mullis and Falkinham, 2013; Rindi and Garzelli, 2014). In the United States, the overall prevalence of infection is estimated to be between 1.4 and 13.9 per 100,000 persons and is increasing by 2.5–8% annually, although the prevalence varies by region, sex, and race/ethnicity (Adjemian et al., 2018). *Mycobacterium avium* complex can cause a range of lung diseases, depending on the demographics and lung status of the infected host. For example, older white males with a history of smoking and underlying chronic obstructive pulmonary disease tend to develop cavitary lung disease, which can resemble tuberculosis clinically and radiographically, with cough, weight loss, and upper lobe infiltrates and cavities (Teirstein et al., 1990). Multiple small lung nodules and bronchiectasis due to MAC have been described in nonsmoking women over the age of 50, who tend to be taller, leaner, and more likely to have pectus excavatum, scoliosis, and mitral valve prolapse than control subjects (Iseman et al., 1991; Kim et al., 2008).

Due to the length, complexity, and toxicity of the treatment regimen, careful consideration must be given to whether treatment should be initiated. If treatment is indicated, the next clinical decisions regard selection of antibiotics and duration of administration. Although patients with noncavitary disease may be monitored initially, it is recommended that those with cavitary disease begin therapy at time of diagnosis, given their risk for rapid progression and irreversible lung damage (Griffith et al., 2007; Daley et al., 2020). Generally, MAC is susceptible to macrolides, aminoglycosides, and clofazimine, with variable susceptibility to rifamycins, fluoroquinolones, ethambutol, and linezolid. Treatment of lung disease due to macrolide-susceptible MAC requires a minimum of three antibiotics, typically consisting of azithromycin (250–500 mg), rifampin (600 mg), and ethambutol (15 mg/kg), given three times weekly for mild or moderate noncavitary nodular bronchiectatic disease, or daily for cavitary or severe nodular bronchiectatic disease. In addition, given the poor prognosis, patients with radiographic presence of cavities may additionally receive parenteral streptomycin or amikacin (10–15 mg/kg three times per week) during the first 8–12 weeks of therapy. Treatment is continued until consecutive sputum cultures are negative for at least 12 months. Since sputum conversion to negative usually takes 3–6 months, a typical patient is often treated for 15–18 months (Griffith, 2007; Ito et al., 2012; Gochi et al., 2015; Daley et al., 2020).

In this review, we will discuss the biological basis for the current complex and lengthy treatment for MAC disease, focusing on the topic of antibiotic tolerance. First, we will define bacterial antibiotic tolerance and persistence and contrast these phenomena with antibiotic resistance. Next, we will discuss the clinical pathology associated with MAC lung disease, and the host microenvironments that may contribute to biofilm formation and antibiotic-tolerant bacteria, as well as clinically relevant *in vitro* and *in vivo* models of MAC infection. We will then review general molecular mechanisms underlying bacterial antibiotic tolerance, particularly those described in MAC. Due to the limited amount of research on antibiotic tolerance in MAC, we will also review relevant mechanisms from related mycobacteria, including *M. tuberculosis*, with a focus on genes containing significant sequence homology in MAC, to identify potential mechanisms warranting further exploration in MAC.

## Antibiotic Tolerance and Persistence vs. Resistance in Bacterial Populations

Antibiotic resistance in bacteria is mediated by genetic mutations in the bacterial chromosome or extrachromosomal plasmids that confer the irreversible ability to survive antibiotic exposure (Kim and Wood, 2017). These genetic mutations are passed on to daughter cells, enabling their continued growth in the presence of antibiotics. Examples of antibiotic resistance mechanisms include: (i) alteration of bacterial proteins representing antibiotic targets; (ii) enzymatic degradation of antibiotics; (iii) changes in membrane permeability (i.e., reduced entry of antibiotics); (iv) increased efflux of antibiotics; (v) alterations of antibiotic-activating enzymes; and (vi) activation of resistant metabolic pathways (Blair et al., 2015).

Although the term “persistence” is often used in the vernacular sense in the literature to refer to prolonged bacterial survival under stress conditions *in vitro* or *in vivo*, historically, microbial persistence is defined as the “capacity of drug-susceptible organisms to survive drug attack when subsisting in an animal body” (McDermott, 1958). More recently, Kim and Wood proposed the following working definition of persisters as “those cells that remain after the actively respiring cells are vanquished by antibiotics” (Kim and Wood, 2016). Although it has been suggested that bacterial persisters are not dormant (Orman and Brynildsen, 2013), the preponderance of evidence indicates reduced metabolism in these cells (Hobby et al., 1942; Bigger, 1944; Shah et al., 2006; Kwan et al., 2013). Single-cell experiments by Balaban et al. (2004) reported that persister cells are generated spontaneously or stochastically in an exponentially growing bacterial population, without antibiotic or other stress exposure (Brauner et al., 2016).

Antibiotic “tolerance” was coined by Tomasz et al. (1970) to refer to the reduced susceptibility of slowly growing or non-growing bacteria to antibiotics, particularly those targeting cell wall synthesis, without a change in the minimum inhibitory concentration (MIC) (Tuomanen et al., 1986). Whether there is a true difference between persistence and tolerance in bacteria remains unclear. Balaban et al. (2004) has suggested that persisters represent small bacterial subpopulations, whereas antibiotic tolerance, resulting from exposure to growth-limiting stresses, is an attribute of the entire bacterial population (Brauner et al., 2016). However, other investigators have claimed that the distinction between antibiotic tolerance and persistence is “contrived” (Kim and Wood, 2016), as evidenced by several studies showing that persister cells can be generated by exposure to antibiotics and other stresses (Dörr et al., 2010; Möker et al., 2010; Vega et al., 2012; Kwan et al., 2013, 2015; Hu et al., 2015; Van den Bergh et al., 2016). In any case, antibiotic-tolerant bacteria and persisters differ from antibiotic-resistant mutants in that their reduced susceptibility to antibiotics is non-heritable and transient, i.e., reversible. Relevant to MAC and other mycobacterial infections, which require combination antibiotic therapy, recent data suggest that if tolerance emerges to one antibiotic, the combination may promote the transmission of resistance to a partner drug (Liu et al., 2020).

## Adaptation to Stress Conditions and the Development of Antibiotic Tolerance

*Mycobacterium avium* complex bacteria encounter a variety of environments and conditions in the infected host, depending on the route of entry and final site of infection. *Mycobacterium avium* complex species enter the respiratory tract and colonize the alveolar space, where they can establish infection within alveolar macrophages or type II alveolar epithelial cells (Bermudez and Goodman, 1996). Similarly, these mycobacteria can enter the gastrointestinal tract through the oral route, and their ability to invade intestinal epithelial mucosal cells is aided by high osmolarity, temperature of 37°C and low oxygen environmental conditions (Kim et al., 1998; Sangari et al., 1999). After phagocytosis by macrophages, MAC can reside within the phagosome, where it is known to inhibit acidification and the expression of proton pumps ATPases, thereby acquiring an antibiotic-tolerant phenotype (Sangari et al., 1999).

*In vitro* models attempting to simulate the microenvironments encountered by MAC in the environment and/or in human host tissues have shown that nutrient starvation, low pH and lack of oxygen induce a nonreplicative state in *M. avium* subsp. *hominissuis* (MAH; Rojony et al., 2019). In response to such stress conditions, *M. avium* alters its transcriptional and metabolic program (Archuleta et al., 2005; Rojony et al., 2019), thus inducing an antibiotic-tolerant phenotype characterized by altered cell wall membrane permeability (Inderlied et al., 1993) and increased expression of efflux pumps (Rodrigues et al., 2009).

*Mycobacterium intracellulare* and *M. avium* exhibit a biphasic response to nutrient starvation, characterized by an adaptive phase of 4–7 days, during which there is a 50% reduction in bacterial viability, followed by a persistence phase, in which the bacteria become metabolically dormant (Archuleta et al., 2005). During the adaptive phase, antibiotic susceptibility already begins to decline concomitantly with increased lipid catabolism, reduced cell wall permeability associated with altered mycolate modifications, loss of catalase and urease activities, reductions in protein levels of alanine tRNA synthetase, and increases in ribonuclease E levels. During the persistence phase, there is dramatically reduced metabolism, with a shift to the glyoxylate shunt, stabilization of the mycolate pool, and a switch to transcription of only essential genes (Archuleta et al., 2005). Importantly, the glyoxylate shunt enzyme isocitrate lyase has been shown to play a key role in long-term survival of *M. tuberculosis* in host tissues (McKinney et al., 2000). These alterations in lipid metabolism could also affect entry of antibiotics into the mycobacteria. The thick, lipid-rich cell wall of mycobacteria plays an important role in restricting penetration of antimicrobial agents (Rastogi et al., 1981; McNeil and Brennan, 1991; Inderlied et al., 1993). Under starvation conditions, changes in cell wall composition could result in a further reduction in antibiotic permeability, thereby contributing to phenotypic tolerance. However, this area remains under investigation (Archuleta et al., 2005).

In order to identify metabolic changes associated with antibiotic tolerance in MAC, Rojony et al. (2019) used Tandem Mass Tag Mass Spectrometry sequencing to quantify proteins in MAH exposed to amikacin (4 μg/ml) and clarithromycin (16 μg/ml) for 24 h under aerobic, anaerobic, and biofilm conditions. The latter two conditions were associated with *de novo* synthesis of proteins involved in pantothenate and CoA biosynthesis, glycerolipid metabolism, nitrogen metabolism, and chloroalkene degradation. The MAH genes *panC* and *panD* are homologous to the eponymous *M. tuberculosis* genes, which are involved in *de novo* biosynthesis of pantothenate (Zheng and Blanchard, 2001), and are required for virulence in an immunocompromised mouse model (Sambandamurthy et al., 2002). The study by Rojony et al. (2019) also showed increased abundance of MAH nitrate, nitrate transporter and nitrate reductive enzymes under anaerobic and biofilm conditions. In *M. tuberculosis*, nitrate has often been shown to replace oxygen as the final electron acceptor l under hypoxic conditions (Gouzy et al., 2014). Chloralkene degradation produces substrates for the glyoxylate shunt (Serafini et al., 2019). In *M. tuberculosis*, it has been shown that glyoxylate can be used in a reductive amination pathway to produce NAD and can be used as an alternative energy source in a nonreplicative state and under anaerobic conditions (Wayne and Lin, 1982; Wayne and Hayes, 1996). Following antibiotic exposure, the common pathways found to be induced in anaerobic and biofilm conditions included peptidoglycan biosynthesis, glycerophospholipid metabolism and protein export (Rojony et al., 2019). Overexpression of the LprB lipoprotein, which is highly synthesized in MAH biofilms during antibiotic exposure, conferred increased antibiotic tolerance relative to the wild-type strain (Rojony et al., 2019).

## Biofilm Formation

The formation of biofilms has been linked to reduced antibiotic susceptibility in bacterial communities due to reduced drug penetration and altered cellular metabolism (Mah, 2012). For example, biofilms formed by *Pseudomonas aeruginosa* in patients with cystic fibrosis showed markedly reduced susceptibility to tobramycin, at least in part due to the production of periplasmic glucan molecules, which inhibit aminoglycoside penetration (Sadovskaya et al., 2010). There is still much debate in the *Mycobacterium* community regarding whether biofilms are formed in host tissues during infection, and whether they contribute to antibiotic tolerance. A study by Yamazaki et al. (2006a) showed that *M. avium* mutants deficient in biofilm formation showed an impaired ability to invade human bronchial epithelial cells and to cause infection in mice (Yamazaki et al., 2006a).

In vitro biofilm studies revealed that extracellular DNA, a key component of the structural integrity of MAH biofilms, plays a role in antibiotic tolerance. Thus, DNase treatment significantly increased the bactericidal activity of moxifloxacin and clarithromycin against MAH in biofilms (Rose et al., 2015). Using a forward genetic screen in MAH, Yamazaki et al. (2006b) identified several enzymes and pathways involved in biofilm formation, including glycopeptidolipid (GPL) and GDP-mannose biosynthesis pathways, 6-oxodehydrogenase (SucA), enzymes of the TCA cycle, protein synthetase (pstB), and the membrane protein, Rv1565c (Yamazaki et al., 2006b). Transcriptional data of MAH biofilms largely corroborated the genetic screen, revealing upregulation of genes encoding enzymes involved in biosynthesis of GPL (e.g., *gtf*) and GDP-mannose (e.g., *guaB2*, *pmmB2*, and *pstB*), components of the TCA cycle (e.g., *accA2* and *accD2*), and those involved in fatty acid biosynthesis (e.g., *Itp3* and *pks10*).

In *M. tuberculosis*, GDP-mannose has been found to be an important modulator of the immune response, acting as a lipid anchor of lipoarabinomannan (Schlesinger et al., 1996) and a ligand between bacteria and phagocytic cells. It is possible that upregulation of such cell wall-associated proteins reduces the permeability of the mycobacterial cell wall to antibiotics, although potential role for these biofilm-forming proteins in antibiotic tolerance remains to be elucidated.

## Mechanisms of Antibiotic Tolerance in MAC

The molecular mechanisms underlying antibiotic tolerance in MAC remain largely undefined. In contrast, certain genetic pathways responsible for this phenomenon have been identified in other bacteria, and, in particular, in *M. tuberculosis*. Several of these genes share significant homology with annotated genes in the genome of MAC species. Below, we discuss regulatory and metabolic pathways implicated in antibiotic tolerance in mycobacteria, and highlight those with MAC homologs using the Mycobacterial Homology Database, which compares genomic sequences across various laboratory strains of mycobacteria (Matern et al., 2018).

### The Stringent Response

One of the key mechanisms implicated in mycobacterial persistence and antibiotic tolerance is the stringent response, which is triggered by nutrient starvation and other stresses and mediated by the rapid accumulation of the alarmone, hyperphosphorylated guanosine ((p)ppGpp) (Boutte and Crosson, 2013). Binding of (p)ppGpp changes the sigma factor specificity of the RNA polymerase, allowing the binding of alternative sigma factors, which have unique promoter recognition and activity (Artsimovitch et al., 2004; Perederina et al., 2004). The mycobacterial stringent response appears to be a critical regulatory pathway driving antibiotic tolerance (Klinkenberg et al., 2010; Thayil et al., 2011; Chuang et al., 2013, 2015). Several genes in *M. avium* bear substantial homology to genes encoding components of the *M. tuberculosis* stringent response, suggesting conservation of this antibiotic tolerance mechanism across mycobacterial species. The *rel*_Mtb_ gene (*rv2583*) is highly homologous to the corresponding genes from the two *M. avium* annotated genomes [93% with gene *DFS55_09310* from strain MAV109 (Matern et al., 2018) and 100% homology with *MAV_3464* from MAV104 (Horan et al., 2006)]. The *M. tuberculosis* genes *rv2984* and *rv3232c*, which encode the polyphosphate kinases, PPK1 and PPK2, respectively, show 80–86% homology to the corresponding genes in MAV 109 (*DFS55_07455* and *DFS55_0547*) and 104 (*MAV_3834* and *MAV_4195*). Likewise, the exopolyphosphatase gene *ppx1* (*M. tuberculosis rv0496*) has ∼85% homology to MAV104 gene *DFS55-03160* and MAV109 gene *MAV_4656*. Lastly, the *M. tuberculosis* exopolyphosphatase gene *ppx2*, *rv1026*, has 78% homology to the MAV109 gene *DFS55_19565* and MAV104 gene *MAV_1167*. The substantial homology between *M. tuberculosis* and *M. avium* genes encoding key components of the stringent response suggest that these genes serve a similar function. However, targeted mutagenesis studies in MAC species are warranted to directly address this hypothesis in the future.

Studies in *M. tuberculosis* have demonstrated that the stringent response enzyme, Rel_Mtb_, plays an important role in antibiotic tolerance. Rel_Mtb_ is a dual-functioning enzyme which is able to synthesize and hydrolyze (p)ppGpp (Lindall et al., 1967; Avarbock et al., 2000). *M. tuberculosis* lacking Rel_Mtb_ (*rv2583*) is unable to induce the stringent response and is defective in long-term survival in nutrient starvation and progressive hypoxia *in vitro* (Primm et al., 2000), as well as in mouse lungs (Dahl et al., 2003). Rel_Mtb_ deficiency leads to defective *M. tuberculosis* survival in a mouse hypoxic granuloma model of latent TB infection (Karakousis et al., 2004a,b) and in guinea pig lungs (Klinkenberg et al., 2010). Recently, Dutta et al. (2019) found that a Rel_Mtb_-deficient *M. tuberculosis* mutant was ∼500-fold more susceptible to killing by the first-line anti-TB drug isoniazid during nutrient starvation and in the lungs of chronically infected mice. A small molecule Rel_Mtb_ inhibitor derived from a >2-million compound screen showed significant activity against nutrient-starved *M. tuberculosis*, as well as synergy with isoniazid (Dutta et al., 2019), which has limited efficacy against *M. tuberculosis* persisters (Dutta and Karakousis, 2014). Given these findings in *M. tuberculosis*, the homologous stringent response genes in *M. avium* may similarly contribute to antibiotic tolerance in the latter species.

### Efflux Pumps

Efflux pumps can contribute to antibiotic resistance by a genetic mutation resulting in dysregulation of pump expression. Recent studies have shown that efflux pumps also play a role in antibiotic tolerance through upregulation of expression, without mutation(s) in the encoding gene. In a study of clinical isolates of *M. avium* and *M. intracellulare* from AIDS patients, susceptibility to the macrolides clarithromycin and erythromycin was increased in the presence of efflux pump inhibitors, such as thioridazine, chlorpromazine, and verapamil (Rodrigues et al., 2009). Collectively, these data suggest that efflux pump inhibitors may serve as adjunctive therapy to target intracellular and extracellular antibiotic-tolerant mycobacteria, rendering them more susceptible to killing by antibiotics. In particular, pumps encoded by MAV_3306 and MAV_1406, which have 91 and 69% homology to *M. tuberculosis* efflux pump genes *rv1473* and *rv1258c* (Schmalstieg et al., 2012; Matern et al., 2018), could be targeted as adjunctive therapy, as has been done in *M. tuberculosis* (Gupta et al., 2013).

RNA expression studies have found increased expression of various efflux pumps in *M. tuberculosis* upon exposure to antibiotics, possibly contributing to antibiotic tolerance. Isoniazid exposure induces expression of *rv2459*, *bacA* (*rv1819c*), *rv3728*, *mmr* (*rv3065*), *efpA* (*rv2846*), *mmpL7* (*rv2942*), *p55* (*rv1410c*), and the tap-like gene *rv1258c*. These genes encode transporters or probable proteins belonging to the ATP binding cassette (ABC) family, major facilitator superfamily, or small multidrug resistance family (Gupta et al., 2010; Kapopoulou et al., 2011; Rodrigues et al., 2011). Antibiotic exposure selects for the population of bacteria with the ability to upregulate these pumps in response to stress conditions. Efflux pump-induced tolerance can also result from mycobacterial adaptation to phagocytosis by macrophages. The percentage of antibiotic-tolerant bacteria was found to be positively correlated to the time spent within macrophages in *Mycobacterium marinum*, a model for mycobacterial infection in zebrafish (Adams et al., 2011). Adams et al. also found that *M. tuberculosis* cultured in macrophages for 96 h had a greater than two-fold increase in surviving bacteria tolerant to both rifampin and isoniazid compared to those cultured intracellularly for only 2 h. These tolerant bacteria were found to have upregulated expression of efflux pumps and their regulatory genes. Tap-like gene *rv1258c* was found to be induced by macrophage infection with rifampin exposure at concentrations below the MIC.

When strains of *M. tuberculosis* were exposed to isoniazid to induce expression of efflux pumps, administration of the calcium channel blocker and efflux pump inhibitor, verapamil, in conjunction with antibiotics reversed the antibiotic-tolerant phenotype, returning MIC values to parental strain levels in vitro. *M. marinum*-infected macrophages had a lower intrabacillary burden following exposure to verapamil in addition to antibiotics (Adams et al., 2011; Rodrigues et al., 2011). In addition, administration of verapamil plus antibiotics reduced the number of lesions and bacillary burden in the lungs of *M. tuberculosis-*infected C3HeB/FeJ mice following 3 months of treatment compared to antibiotic treatment alone (Gupta et al., 2013). These results indicate a potential use of these inhibitors as adjunctive therapy in MAC infection to counteract tolerance induced by efflux pumps.

### PhoU and Phosphate-Sensing Pathways

PhoU, initially identified as conferring antibiotic tolerance in *Escherichia coli*, shares homology with other proteins in many bacterial species, including *M. tuberculosis* (Li and Zhang, 2007). The homologs, *phoY1* (*rv3301c*) and *phoY2* (*rv0821c*), have 40 and 44% sequence homology to *E.coli phoU*, respectively, and also appear to contribute to *M. tuberculosis* tolerance to antibiotics (Shi and Zhang, 2010). In *M. tuberculosis*, the PhoY proteins function to negatively regulate the two-component regulatory system SenX3-RegX3 system (Namugenyi et al., 2017), which is responsible for sensing and responding to inorganic phosphate limitation (Glover et al., 2007; Rifat et al., 2009, 2014; Rifat and Karakousis, 2014). In addition, *phoY2* was found to be significantly upregulated in *M. tuberculosis* after 96 h of nutrient starvation (Betts et al., 2002). A double knockout of the PhoY proteins conferred a loss of the persistence phenotype both in vitro and in vivo after antibiotic exposure (Namugenyi et al., 2017). In a separate study, a recombinant strain of *M. tuberculosis* deficient in PhoY2 showed increased susceptibility to rifampin and pyrazinamide, with reduced MIC and MBC of rifampin (Shi and Zhang, 2010). A *phoY2*-deficient mutant, but not a *phoY1*-deficient mutant, showed reduced survival in the lungs and spleens relative to the complemented strain. These results indicate that disruption of the phosphate sensing system, specifically of phoY2, is important for antibiotic tolerance in *M. tuberculosis*. The *M. avium* genome contains a *phoU* gene (*MAV_0768*), which is 94% homologous to *M. tuberculosis phoY2* (*rv0821c*). Further studies are required to elucidate the potential role of *MAV_0768* in antibiotic tolerance of MAC species.

## Conclusion

Mycobacteria are among the oldest known human pathogens, yet they continue to take a major toll on global health. Infections due to mycobacteria require complicated and lengthy antibiotic regimens, which pose challenges for medical adherence, thereby contributing to the emergence of antibiotic resistance. In order to develop novel, abbreviated therapies, a deeper understanding of the mechanisms of antibiotic tolerance in mycobacteria is needed.

Like other bacteria, MAC displays reduced susceptibility to antibiotics within biofilms. In theory, degradation of the extracellular matrix of biofilms by hydrolytic enzymes and other bioactive agents could improve antibiotic penetration and reverse the metabolic downshift of the mycobacteria within biofilms, rendering them more susceptible to killing by conventional antibiotics (Koo et al., 2017). However, further studies are needed first to determine what role, if any, biofilms play in establishment of chronic MAC infection and antibiotic tolerance in human lungs and other tissues. Drug efflux pumps appear to play a role in antibiotic tolerance in MAC species and other mycobacteria. Given the available evidence, we believe that clinical studies of centrally acting calcium channel blockers, such as verapamil, and other efflux pump inhibitors are warranted to determine if these agents can serve as adjunctive therapy for lung disease due to MAC.

In this review article, we have highlighted the *M. avium* homologs of various *M. tuberculosis* factors, which have been implicated in antibiotic tolerance, including the stringent response and phosphate-sensing pathways. Recombinant strains of MAC deficient in these factors/pathways should be generated to determine their role in antibiotic tolerance under various in vitro stress conditions and in relevant animal models. Specifically, an inducible CRISPR interference (CRISPRi) system (Larson et al., 2013; Rock et al., 2017) can be used to “knock down” expression of putative tolerance genes, as this technique may more closely simulate pharmacological inhibition of the corresponding gene products. In addition to these targeted approaches, forward genetic screens using MAC transposon mutant libraries exposed to sub-lethal concentrations of antibiotics under stress conditions can be used to identify novel genes/pathways responsible for antibiotic tolerance in an unbiased manner. The identification of MAC “antibiotic tolerance factors” would pave the way for compound library screens to identify small molecule inhibitors of these factors. Candidate compounds could then be tested as adjunctive therapies to determine whether they render tolerant organisms more susceptible to killing by standard antibiotics, with the goal of shortening treatment for MAC and other mycobacterial infections to improve patient outcomes.

## Author Contributions

HP and PK designed and directed the project. HP, RL, and JR performed the literature search. All authors wrote the article.

## Conflict of Interest

The authors declare that the research was conducted in the absence of any commercial or financial relationships that could be construed as a potential conflict of interest.
